# Evaluating the Efficacy of a Novel Side-Support Surgical Tray Stand for Endoscopic Transnasal Skull Base Surgery: A Prospective Study

**DOI:** 10.7759/cureus.50987

**Published:** 2023-12-23

**Authors:** Jing Zhang, Xiaonan Liu, Wei Wang, Songbai Gui, Lei Cao

**Affiliations:** 1 Nursing, Beijing Tiantan Hospital, Capital Medical University, Beijing, CHN; 2 Neurosurgery, Beijing Tiantan Hospital, Capital Medical University, Beijing, CHN

**Keywords:** operating room nursing, satisfaction, infection, upright, tray stand

## Abstract

Objective

Endoscopic transnasal skull base surgery is a valuable technique used in the surgical treatment of various skull base pathologies. In such surgeries, the reconstruction of the skull base is crucial for surgical success and minimizing complications. This study presents a new side-support surgical tray designed to improve the exposure of the lateral femoral surgical area during surgery, enhancing surgical efficiency and reducing the risk of surgical complications. The study compared this innovative tray stand with the conventional double-sided support tray stand to evaluate its impact on surgical procedures and complications.

Materials and methods

The study prospectively analyzed 248 endoscopic transnasal skull base surgeries requiring lateral femoral autologous tissue harvesting. One hundred fifty-eight cases were performed using the side-support surgical tray stand (experimental group), while 90 cases used the conventional double-sided support tray stand (control group). Various parameters were evaluated, including satisfaction scores of surgeons, circulating nurses, instrument nurses, and anesthetists, as well as objective outcomes such as surgical duration and the incidence of complications.

Results

Surgeons in the experimental group expressed higher satisfaction with the surgical field exposure and the portability of the surgical tray stand compared to the control group. Likewise, circulating nurses in the experimental group reported greater satisfaction with the installation and portability, surpassing that of the control group (*p*< 0.01). Although the stability of instrument nurses in the experimental group was slightly less than that of the control group, it had no discernible impact on surgical cooperation. Anaesthesiologists in the experimental group exhibited higher satisfaction regarding the convenience of intraoperative monitoring and management than their counterparts in the control group. The average duration required for intraoperative autologous tissue harvesting in the experimental group was significantly shorter than in the control group (*p* < 0.01). Furthermore, the incidence of postoperative wound infections and intracranial infections in the experimental group was notably lower than in the control group (would infections, *p* = 0.046; intracranial infection, *p* = 0.025).

Conclusion

The novel side-support surgical tray stand effectively improves surgical exposure, convenience, and safety while reducing the risk of surgical site and intracranial infections. It also shortens surgical duration and lowers complication rates, making it a suitable choice for clinical application.

## Introduction

With the advancement of endoscopic techniques, endoscopic transnasal skull base surgery has gained widespread application in the surgical management of skull base conditions such as pituitary adenomas, craniopharyngiomas, and sellar region meningiomas [1-3]. These procedures often necessitate the intentional or inadvertent opening of the skull base dura mater, resulting in communication between intracranial and extracranial spaces. The success of skull base reconstruction plays a pivotal role in influencing surgical outcomes and mitigating the risk of surgical complications. Currently, materials employed for intraoperative skull base reconstruction encompass pedicled or free autologous tissues and synthetic materials [4]. Among the free autologous tissues used, muscle, fascia, and fat are common, and the lateral femoral is a convenient donor site, widely utilized due to its accessibility and capacity for larger tissue harvests [4,5].

The conventional surgical tray stand typically employs a double-sided configuration affixed to the operating table, with support rods situated laterally at the thigh level, obstructing the surgical field. This arrangement results in autologous tissue harvest being significantly inconvenient, increases procedural complexity, and poses a risk of surgical site infections. Furthermore, it can lead to the contamination of autologous tissues and elevate the chances of intracranial infections. Conventional surgical tray stands may clash with the base of the operating table, making it unfeasible to secure the surgical trays above the abdominal area. In an instance encountered by the authors, during the harvest of lateral thigh fascia for repair, the patient developed thigh wound infections, intracranial infections, and severe complications, including a liver abscess.

Consequently, an imperative clinical demand exists for a tray stand that can be attached to one side of the operating table to facilitate the exposure of the femoral surgical area during surgery. This approach enhances surgical efficiency and diminishes the risk of surgical site and intracranial infections. To address this need, the present study designed a side-support surgical tray stand with the objective of streamlining the intraoperative exposure of the lateral femoral surgical area. The study endeavors to evaluate its impact on surgical procedures and complications.

## Materials and methods

Patients and surgeries

Between October 2019 and October 2020, 920 endoscopic transnasal skull base surgeries were performed in the Department of Neurosurgery at Beijing Tiantan Hospital, Capital Medical University. This prospective study selected 248 cases that necessitated intraoperative harvesting of lateral femoral autologous tissues as study subjects. The study received ethical approval from the Ethics Committee of Beijing Tiantan Hospital, Capital Medical University (KY 2020-084-02), and all patients provided informed consent.

Design of the side-support surgical tray stand

**Figure 1:**
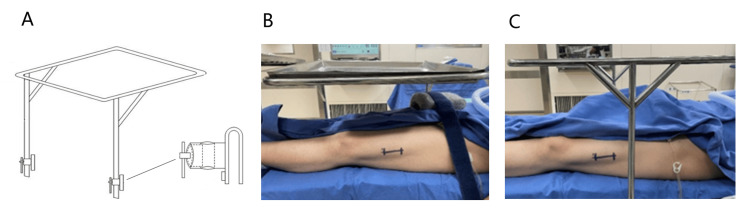
Schematic diagram of the side-support surgical tray stand and comparison of surgical field exposure with the conventional double-sided support tray stand The side-support surgical tray stand is constructed with two fixed support rods positioned on the same side of the operating table (A). In contrast to the conventional double-sided support tray stand (C), the side-support tray stand (B) provides unobstructed surgical field exposure, resulting in enhanced exposure.

The conventional surgical tray stands feature support rods on both sides, positioned laterally on the operating table. In contrast, the side-support surgical tray situates the frame on one side, utilizing triangular pillars on both sides to support the tray. A specialized table-fixing device secures it to the operating table. This configuration permits the tray to be effortlessly rotated along with the operating table during the procedure without exerting pressure on the patient's body. The design of the side-support tray stand aims to enhance the exposure of the lateral femoral surgical area and mitigate interference caused by the support rods of the conventional tray stand (Figure 1).

Satisfaction evaluation

A quantitative assessment of the surgeon, circulating nurse, instrument nurse, and anesthetist's satisfaction with using the two surgical tray stands was conducted using a scoring system (Table 1). In total, 10 neurosurgeons, 82 nurses, and 42 anesthetists participated in this survey. Surgeon satisfaction encompassed the surgical field exposure, the convenience of draping and the surgical process, and willingness to use the two trays. Circulating nurses assessed the ease and safety of tray stand installation. Instrument nurses evaluated tray stand stability and its influence on intraoperative instrument coordination. Anesthetists gauged tray stand convenience for intraoperative anesthesia management and their willingness to use it. They completed the questionnaires immediately when the surgeries finished. The scoring system employed a 5-point Likert scale with 15 items (5 = very satisfied, 4 = satisfied, 3 = somewhat satisfied, 2 = dissatisfied, 1 = very dissatisfied). Each item demonstrated a content validity index (CVI) ranging from 0.80 to 1.00, with an average CVI of 0.95. A preliminary survey involving 15 patients produced an overall Cronbach's alpha coefficient of 0.834.

**Table 1:** Questions of the satisfaction scale on the support tray stands

Questionnaire						
Patient No.		Support tray				
Surgeons		Very satisfied	Satisfied	Somewhat satisfied	Dissatisfied	Very dissatisfied
1. How satisfied or dissatisfied are you with the support tray?						
2. How satisfied or dissatisfied are you with the exposure of the surgical field?						
3. How satisfied or dissatisfied are you with the convenience of draping and the surgical process?						
4. How satisfied or dissatisfied are you with willing to use the tray						
Circulating nurses		Very satisfied	Satisfied	Somewhat satisfied	Dissatisfied	Very dissatisfied
1. How satisfied or dissatisfied are you with the support tray?						
2. How satisfied or dissatisfied are you with the convenience of tray stand installation?						
3. How satisfied or dissatisfied are you with the safety of tray stand installation?						
4. How satisfied or dissatisfied are you with willing to use the tray						
Instrument nurses		Very satisfied	Satisfied	Somewhat satisfied	Dissatisfied	Very dissatisfied
1. How satisfied or dissatisfied are you with the support tray?						
2. How satisfied or dissatisfied are you with the stability of the tray stand?						
3. How satisfied or dissatisfied are you with the tray’s influence on intraoperative instrument coordination?						
4. How satisfied or dissatisfied are you with willing to use the tray						
Anaesthetists		Very satisfied	Satisfied	Somewhat satisfied	Dissatisfied	Very dissatisfied
1. How satisfied or dissatisfied are you with the support tray?						
2. How satisfied or dissatisfied are you with the tray stand convenience for intraoperative anaesthesia management?						
3. How satisfied or dissatisfied are you with the convenience for intraoperative handling?						

Surgical outcome evaluation

Recorded data, including the time taken for both groups of surgeries, along with the incidence of wound infections, intracranial infections, subcutaneous hematomas, and other surgical complications. Comparative analyses were performed to identify differences between the two groups.

Statistical Analysis

Data analysis was executed using SPSS 17.0 statistical software(Chicago: SPSS Inc). Normally distributed continuous data were presented as mean ± standard deviation (x̄ ± s) and analyzed between groups using t-tests. Count data were conveyed as frequencies or percentages and compared between groups using chi-square tests. Pearson correlation analysis was performed between the usage of surgical tray stands and the satisfaction scores of the medical workers. A significance level of p < 0.05 indicated statistical significance.

## Results

Clinical data of the patients

The experimental group utilized the new side-support surgical tray stand, consisting of 158 cases (76 males and 82 females), with an average age of 42.3 ± 8.3 years. This group comprised 92 subdural approach surgeries and 66 extradural approach surgeries. The control group employed the conventional double-sided support surgical tray stand, encompassing 90 cases (41 males and 49 females), with an average age of 40.9 ± 7.5 years, including 50 subdural approach surgeries and 40 extradural approach surgeries. There were 65 pituitary adenomas, 50 craniopharyngiomas,15 chordomas, 26 sellar tuberculum meningiomas, 2 optic pathway gliomas in the experiment group, while 36 pituitary adenomas, 32 craniopharyngiomas, 8 chordomas, 2 sellar tuberculum meningiomas, 2 germinomas in the control group. All these patients required strengthened skull base repairment to detect intraoperative cerebrospinal fluid leaks. The fat plus fascia was used to repair the defects. No significant difference was found between the two groups in these clinical data.

The novel surgical tray was satisfied by surgeons, nurses, and anesthetists

Statistical analyses were conducted to assess the surgeon, circulating nurse, instrument nurse, and anesthetist's satisfaction with the two surgical tray stand types. The results revealed that surgeon satisfaction with the side-support surgical tray stand was significantly higher than that of the control group, primarily regarding surgical field exposure (p < 0.01) and procedural convenience (p < 0.01). Surgeons also expressed a significantly greater willingness to use the side-support tray stand than the control group (p < 0.01). The satisfaction scores of surgeons were strongly positively related to the novel support tray stand and surgical field exposure, surgical convenience, and willingness to use (Pearson correlation coefficient, 0.883; 0.830; 0.835). Circulating nurses reported increased satisfaction with the side-support surgical tray stand, primarily due to the convenience of its installation, compared to the control group (p < 0.01). There was no significant difference in terms of safety between the two groups. The satisfaction scores of circulating nurses were positively related between the novel support tray stand and installation convenience (Pearson correlation coefficient, 0.553). Instrument nurses perceived slightly lower stability in the side-support tray stand than the control group, but this was considered acceptable and did not affect intraoperative coordination (t-test,p = 0.43; Pearson correlation coefficient, -0.81 ). Anesthetists found the side-support tray stand significantly more convenient for intraoperative anesthesia management than the control group (p < 0.01). The satisfaction scores of anesthesiologists were positively related between the novel support tray stand and intraoperative monitoring and handling convenience (Pearson correlation coefficient, 0.548, 0.659) (Table 2).

**Table 2:** Satisfaction of surgeons, nurses, and anesthetists on the two surgical tray stands

Groups	Surgeons			Circulating Nurses		Instrument Nurses		Anesthestists	
	Surgical Field Exposure	Surgical Convenience	Willingness to Use	Installation Convenience	Safety	Stability	Intraoperative cooperation	Intraoperative Monitoring Convenience	Intraoperative Handling Convenience
Experimental group (158)	4.78±0.51	4.32±0.45	4.82±0.45	4.58±0.75	4.64±0.65	3.86±0.78	4.51±0.67	4.34±0.55	4.45±0.67
Control group (90)	2.37±0.78	2.41±0.83	2.85±0.88	3.32±1.15	4.75±0.57	4.58±0.69	4.58±0.70	3.42±0.85	3.15±0.81
t-value	29.1	25.4	26.7	10.51	-1.42	-7.28	-7.97	11.23	14.64
*p*-value	＜0.01	＜0.01	＜0.01	＜0.01	0.158	＜0.01	0.43	＜0.01	＜0.01
Pearson correlation coefficient	0.883	0.830	0.835	0.553	-0.81	-0.421	-0.51	0.548	0.659
*p*-value	＜0.01	＜0.01	＜0.01	＜0.01	0.202	＜0.01	0.426	＜0.01	＜0.01

Novel surgical trays reduced surgical complications

A comparative analysis of the two groups included the surgeries' duration and complications' incidence. The results indicated that the surgical duration of femoral autologous tissue harvest in the experimental group was significantly shorter than in the control group (p < 0.01). The overall incidence of complications in the experimental group was notably lower than in the control group, with no significant differences observed in subcutaneous hematomas (p = 0.32) and wound infections (p = 0.14). However, the incidence of intracranial infection in the experimental group was significantly lower than in the control group (p < 0.01) (Table 3).

**Table 3:** Comparison of the complications of femoral autologous tissue harvest surgeries between two surgical trays

Groups	Surgical Duration (minutes)	Postoperative Wound Infection	Postoperative Hematoma	Postoperative Intracranial Infection
Experimental group (158)	16.6±4.2	0.6% (1/158)	0.6% (1/158)	3.2% (5/158)
Control group (90)	24.7±4.9	5.6% (5/90)	3.3% (3/90)	10% (9/90)
t value /Chi-square	9.24	3.98	1.20	5.02
*p*-value	＜0.01	0.046	0.272	0.025

## Discussion

Necessity of designing a side-support tray stand

Endoscopic transnasal skull base surgery often involves either active or passive opening of the skull base dura, leading to communication between the intracranial and extracranial spaces, thereby increasing the risk of intracranial infections and other complications [6-7]. Properly reconstructing the skull base is critical for reducing postoperative cerebrospinal fluid leaks [8,9]. Currently, autologous tissues are commonly used for skull base reconstruction due to their excellent tissue compatibility, biocompatibility, and high success rates [10]. Frequently, autologous tissues are harvested from the fat, fascia, and/or muscle tissues of the abdomen or thigh [11,12]. Among these sources, the thigh offers a richer supply of autologous materials compared to the abdomen, making it a crucial consideration in endoscopic transnasal skull base surgery.

The placement and manner of surgical tray stands directly influence the coordination of medical staff during the procedure and the convenience of the surgeon's actions [13,14]. Neurosurgical procedures often require surgical trays to be positioned above the patient's chest or abdomen, allowing surgical instrument nurses to efficiently pass instruments [15-17]. Conventional surgical tray stands employ double-sided support rods fixed to the operating table, which are conducive to changes in patient positioning and are widely used in cranial surgeries [17]. However, with the advancement of endoscopic neurosurgery, there is a frequent need to harvest autologous tissues from the patient's thigh during the procedure. The obstructive characteristics of conventional double-sided support tray stand support rods, including blocking the surgical field and the risk of contamination, significantly affect patient outcomes.

Evaluation analysis of medical staff on the side-support tray

Our team designed a side-support surgical tray stand based on endoscopic skull base surgery characteristics. This design involves repositioning the support rod away from the surgical area and utilizing a triangular pillar for the surgical tray stand, maximizing the tray's stability. As a result, the lateral aspect of the patient's thigh is free from support rod obstructions, allowing for better exposure to the surgical field and enhanced surgical convenience. Consequently, using the side-support tray stand reduced the time required for intraoperative autologous material harvesting from an average of 24.7±4.9 minutes with the double-sided support tray stand to 16.6±4.2 minutes, significantly improving surgical efficiency. Conventional tray stands with support rods placed within the surgical area require additional disinfection wrapping of the support rods when draping the surgical field, making the disinfection process and draping of the surgical area quite challenging. The support rods obstruct the surgeon's view and interfere with incisions and suturing during the surgical procedure. In contrast, the new side-support tray stand, without support rods obstructing the surgical field, significantly enhances surgeon satisfaction with the full surgical field exposure and surgical convenience compared to the conventional double-sided support tray stand. Surgeons also expressed a higher willingness to use the side-support tray stand.

Surveying the circulating nurses revealed that the installation convenience of the new tray stand was significantly higher than that of the conventional tray stand. They reported satisfaction with the safety of both options. This is because the conventional double-sided support tray stand, with support rods on both sides of the operating table, requires additional assistance from another healthcare worker for support rod installation, making it difficult for a single person to complete independently. In contrast, the side-support surgical tray stand, with support rods all on one side, can be independently installed and secured by a single circulating nurse, significantly improving installation convenience. Both tray stands are securely fixed to the operating table using dedicated fixtures, ensuring stability without interfering with patient positioning adjustments. Instrument nurses expressed satisfaction with the stability of the new tray stand, although their satisfaction was slightly lower than with the conventional double-sided support tray stand. This is because, in the case of the side-support tray stand, the stability may be slightly compromised if surgical instruments are predominantly on the opposite side of the instrument table. However, this does not significantly affect instrument nurses' coordination during the procedure.

Anesthetists found that the side-support tray stand, with no support rods on the side, allowed for excellent exposure of the patient's chest, abdomen, and same-side upper and lower limbs, facilitating direct patient observation and various intraoperative monitoring and interventions. The new side-support tray significantly improved the convenience of anesthesia management during the procedure, compared to the conventional double-sided support tray stand.

Application of the side-support tray to reduce postoperative complications

Serious complications related to harvesting autologous thigh tissue include wound infection and postoperative hematoma formation [11]. This study compared the two groups regarding surgical complications, and it was observed that using the new side-support tray stand reduced the wound infection rate from 5.6% to 0.6% and decreased the incidence of postoperative hematomas from 3.3% to 0.6%. This reduction was primarily due to the side-support tray stand offering complete exposure to the surgical field, improved illumination in the surgical area, easier hemostasis, and the elimination of support rod contamination in the surgical field, as well as the significant reduction in surgical time. As a result, the risk of postoperative wound infections was substantially reduced, and the occurrence of postoperative hematomas also decreased significantly.

Protecting the harvested autologous tissue from contamination is crucial for reducing the risk of intracranial infections and improving tissue survival, as free autologous tissues inherently have limited infection resistance [18-19]. Compared to conventional tray stands, the new side-support tray stand significantly reduces the occurrence of postoperative intracranial infections in patients undergoing endoscopic transnasal skull base surgery. This is because the new tray stand effectively safeguards the sterile environment of the harvested autologous tissue, lowering the chances of contamination by the tray stand during the tissue harvesting procedure. This, in turn, substantially reduces the incidence of postoperative infections, enhancing the overall surgical outcome for patients.

Limitations

One limitation of this study is the sample size, although sufficient for the reported analyses, may limit the ability to detect rare complications or differences in outcomes between groups. Larger multicentre studies with diverse patient populations could provide a more comprehensive understanding of the generalizability and effectiveness of the side-support surgical tray support. Furthermore, the study primarily assessed subjective satisfaction scores from surgical personnel, and objective measures related to patient outcomes were limited. While satisfaction scores provide valuable insights into the user experience, a more comprehensive analysis incorporating diverse outcome measures could strengthen the study's conclusions.

## Conclusions

The side-support surgical tray stand significantly enhances surgical field exposure and reduces the risk of surgical infections and postoperative hematomas, ensuring safe and efficient surgery. It offers convenience in installation, good safety, and excellent stability. Furthermore, it reduces the incidence of postoperative intracranial infections in patients, making it a valuable addition to endoscopic transnasal skull base surgery that deserves broad application.
